# Abnormal proximal-distal interactions in upper-limb of stroke survivors during object manipulation: A pilot study

**DOI:** 10.3389/fnhum.2022.1022516

**Published:** 2022-11-04

**Authors:** Thanh Phan, Hien Nguyen, Billy C. Vermillion, Derek G. Kamper, Sang Wook Lee

**Affiliations:** ^1^Department of Biomedical Engineering, Catholic University of America, Washington, DC, United States; ^2^Center for Applied Biomechanics and Rehabilitation Research, National Rehabilitation Hospital, Washington, DC, United States; ^3^Department of Internal Medicine, Yale University School of Medicine, New Haven, CT, United States; ^4^UNC/NC State Joint Department of Biomedical Engineering, North Carolina State University, Raleigh, NC, United States; ^5^Department of Physical Medicine and Rehabilitation, Northwestern University, Chicago, IL, United States; ^6^Department of Mechanical Engineering, Korean Advanced Institute of Science and Technology, Daejeon, South Korea

**Keywords:** stroke, upper extremity, abnormal coupling, finger extension, abnormal synergy, intermuscular coherence

## Abstract

Despite its importance, abnormal interactions between the proximal and distal upper extremity muscles of stroke survivors and their impact on functional task performance has not been well described, due in part to the complexity of upper extremity tasks. In this pilot study, we elucidated proximal–distal interactions and their functional impact on stroke survivors by quantitatively delineating how hand and arm movements affect each other across different phases of functional task performance, and how these interactions are influenced by stroke. Fourteen subjects, including nine chronic stroke survivors and five neurologically-intact subjects participated in an experiment involving transport and release of cylindrical objects between locations requiring distinct proximal kinematics. Distal kinematics of stroke survivors, particularly hand opening, were significantly affected by the proximal kinematics, as the hand aperture decreased and the duration of hand opening increased at the locations that requires shoulder abduction and elbow extension. Cocontraction of the extrinsic hand muscles of stroke survivors significantly increased at these locations, where an increase in the intermuscular coherence between distal and proximal muscles was observed. Proximal kinematics of stroke survivors was also affected by the finger extension, but the cocontraction of their proximal muscles did not significantly increase, suggesting the changes in the proximal kinematics were made voluntarily. Our results showed significant proximal-to-distal interactions between finger extension and elbow extension/shoulder abduction of stroke survivors exist during their functional movements. Increased cocontraction of the hand muscles due to increased neural couplings between the distal and proximal muscles appears to be the underlying mechanism.

## Introduction

The human upper extremity is capable of performing a myriad of functional tasks due to its biomechanical structure and its abundant motor and sensory neural innervation. Unfortunately, this dexterity is often compromised by stroke, potentially affecting self-care, employment opportunities, and societal interactions (Cerniauskaite et al., 2012).

While difficulties occurring at the joint or muscle level, such as hypertonicity (Katz and Rymer, 1989), spasticity (Opheim et al., 2014), or weakness (Ada et al., 2003; Kamper et al., 2006) certainly contribute to functional deficits, impairment of the coordination activity of multiple joints may have an even more profound effect (Ada et al., 2006). Past studies examining the arm have revealed altered interjoint kinematics in the paretic arm of stroke survivors (Cirstea et al., 2003) and reduced capacity to create the required joint interaction torques (Beer et al., 2000). Abnormal neurological coupling, as part of a flexion synergy, has also been described between muscles of the elbow and shoulder following stroke (Dewald et al., 1995; Ellis et al., 2009, 2017). These synergies have been shown to extend to finger flexor muscles as well (Miller and Dewald, 2012; McPherson and Dewald, 2019). Indeed, the number of distinct activation patterns available to stroke survivors seems to be reduced (Roh et al., 2012). Increased neural couplings between the distal and proximal muscles could affect the underlying modular structures of the spinal cord that control the arm and hand muscles during functional activities (Cheung et al., 2012).

Object manipulation, a common component of task performance, requires further interjoint coordination as the arm must position, orient, and stabilize the hand while the fingers interact with the external object. Separate neural pathways have been suggested for control of the arm and hand (Galletti et al., 2003; Fattori et al., 2010; Alstermark and Isa, 2012) and the extent of communication between these pathways has been debated (Wing et al., 1996). This coordination is likely impacted by stroke. While grasp-retrieve-release tasks have been widely studied in neurologically intact individuals (Johansson, 1998), they have not been as widely examined after stroke. In particular, the functional impact of the region of the workspace in which the grasp occurs on the hand function of stroke survivors has not been examined. In particular, few studies of this type have combined electromyography (EMG) and kinematic analyses to examine functional movements of the hand and arm following stroke. Because of the high complexity of UE functional tasks (i.e., a large number of DOFs; Valevicius et al., 2019), most kinematic assessments of stroke survivors typically focus on their shoulder and elbow motion, while excluding their finger movements (Schwarz et al., 2019).

In this pilot study, therefore, we aimed to elucidate the impact of abnormal intersegmental interactions of stroke survivors on their function. In particular, we examined in details the kinematics and muscle coordination patterns across all phases of complex functional task performance of chronic stroke survivors and control subjects. In order to overcome aforementioned difficulties, we adopted a within-subject design that varies task conditions (i.e., target locations) to induce systematic changes in proximal kinematics of stroke survivors during functional tasks, thereby allowing us to examine the effects of proximal movements on the distal mechanics of stroke survivors (proximal-to-distal impact) even with the high between-subject variability in their task mechanics. Conversely, distal-to-proximal impact was examined by comparing proximal kinematics during the phases that involved different distal movements (hand open vs. grip) within each subject. We also examined intermuscular coherence between the distal and proximal UE muscles of stroke survivors during task performance to gauge the neural coupling between these muscles, which could indicate underlying mechanisms of functional impairment following stroke (Lemon, 2008). The goal was two-fold; the primary objective was to identify the impact of distal-proximal interactions on task performance. We also aimed to verify if a detailed phase-by-phase analysis can be plausible, given a high between-subject variability of muscle activation and movement kinematics during functional activities (Wagner et al., 2007; Thies et al., 2009).

We expected to observe that the proximal movements of stroke survivors will significantly affect the kinematics and muscle activation of the distal tasks. Here, we anticipated that the abnormal distal-proximal interactions would be detected by a significant increase in the common neural inputs to the distal and proximal muscles. We also expected that the distal movements will also change the proximal kinematics, albeit to a lesser degree, as cortical projection to the arm muscles was found stronger after stroke in comparison to its connections to the hand muscles (Taga et al., 2021).

## Materials and methods

### Subjects

A total of 14 subjects, including nine chronic stroke survivors (S1–S9) and five neurologically-intact control subjects (C1–C5), participated in the study. Three stroke survivors had severe/severe-moderate upper-limb impairments as indicated by their Fugl-Meyer Assessment of Motor Recovery for the Upper Extremity (FMUE) scores (mean ± SD = 29.7 ± 2.9) and six stroke survivors exhibited moderate-mild UE impairments (FMUE mean ± SD = 44.2 ± 1.9) (see Table 1 for stroke subject characteristics), categorized according to a previous study that classified stroke survivors according to their movement impairments (Woytowicz et al., 2017). All five control subjects were right-handed (age: 30 ± 2.5; 2 women).

**TABLE 1:** Stroke subject characteristics.

No.	Age (years)	Sex	Side of hemiplegia	Type of stroke	Time since stroke (years)	FMUE (66)	ARAT (57)	
							Left side	Right side
1	60	Male	Left	Ischemic	10	28	8	47
2	48	Male	Right	Ischemic	21	33	57	9
3	55	Male	Left	Ischemic	7	28	8	57
4	71	Male	Right	Ischemic	30	43	54	48
5	56	Male	Right	Ischemic	4	47	52	47
6	62	Male	Right	Ischemic	10	45	55	37
7	72	Female	Right	Ischemic	3	44	57	48
8	46	Female	Right	Ischemic	5	42	57	27
9	65	Male	Right	Ischemic	3	37	38	57

FMUE, Fugl-Meyer Assessment of Motor Recovery for the Upper Extremity; ARAT: Action Research Arm Test.

The experimental protocol was approved by the Institutional Review Boards of MedStar Health Research Institute, and written informed consent was obtained from each subject prior to participation.

### Instrumentation

An eight-camera motion capture system (Osprey Motion Analysis Corporation) captured joint kinematics with the sampling rate of 60 Hz. Reflective markers were placed on the following bony landmarks: metacarpophalangeal (MCP), proximal interphalangeal (PIP), and distal interphalangeal (DIP) joints of the index and middle fingers, interphalangeal (IP), MCP, and carpometacarpal (CMC) joints of the thumb, radial and ulnar styloid processes, lateral epicondyle of the elbow, acromion process of the shoulder, and torso (lateral ends of the right and left clavicles, and sternum).

Seven pairs of disposable, self-adhesive Ag/AgCl surface electrodes were placed on the following muscles: extensor digitorum communis (EDC), flexor digitorum superficialis (FDS), first dorsal interosseous (FDI), biceps brachii (BB), lateral head of the triceps brachii (TB), anterior deltoid (AD), and lateral deltoid (LD). During the experiment, EMG signals were amplified, band-pass filtered between 5 and 400 Hz, and sampled at 1 kHz (Myosystem 1600A, Noraxon Inc., AZ, USA).

### Experimental protocol

Subjects were first asked to create maximum activations of seven muscles, which were used to normalize EMG for subsequent analyses. Subjects were first asked to perform isometric contraction of the target muscle while placing each electrode so that muscle palpation can be used to identify the muscle location, which were finger extension (EDC), proximal interphalangeal joint flexion (FDS), index finger abduction (FDI), elbow flexion (BB) and extension (TB), and shoulder flexion (AD) and abduction (LD). The same set of tasks were performed for approximately 2 s to record maximum voluntary contraction of each muscle.

**FIGURE 1:**
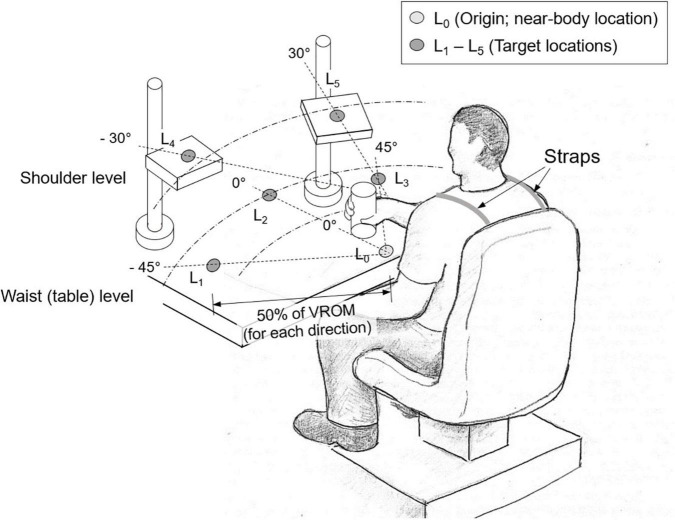
Experimental setup. Subjects were asked to first transport a cylindrical object from the near-body location (L_0_) to one of the target locations (L_1_–L_5_) (“transport” task), which were determined as 50% of their voluntary range of motion (VROM) in each direction. Then they retrieved the object from the target location back to the original location (“retrieve” task). L_1_ and L_4_ were located in the contralateral to the more-impaired side, and L_3_ and L_5_ in the ipsilateral to the more-impaired side.

Subjects then sat in front of a table on which five target locations were marked with blue circles. Subjects were strapped to the chair to prevent compensatory torso movements during reaching. The initial location (L_0_) was marked on the table at waist level in front of the torso and three target locations (L_1_–L_3_) were marked in three different movement directions: L_1_ (45° from midline contralateral to the more-impaired arm), L_2_ (midline), and L_3_ (45° from midline ipsilateral to the more-impaired arm). Two other targets were located at shoulder level by using height-adjustable platforms: L_4_ (30° from midline, contralateral to the more-impaired arm) and L_5_ (30° from midline, ipsilateral to the more-impaired arm) (Figure 1). Reaching each location requires distinct coordination of proximal DOFs. L_1_ mainly requires shoulder adduction and elbow extension; L_2_ mostly elbow extension; L_3_ shoulder abduction and elbow extension; L_4_ shoulder flexion and adduction; and L_5_ shoulder flexion and abduction.

Subjects first performed reaching movements in each of the five movement directions to determine maximum voluntary reaching distance in each direction. Stroke survivors used their more-impaired arm while control subjects used their dominant arm. The target location in each direction was then positioned at 50% of the maximum distance each subject could reach for a given target direction in order to make the task difficulty comparable across subjects with different voluntary ranges of motion.

Once the five target locations were determined, for each of these locations, subjects transported a cylindrical object (diameter: 5 cm, height: 15 cm) from the origin (L_0_) to the target location (“transport” task: T1), released the object, and then returned the hand to the location L_0_. After a brief rest (2–3 s), the subjects repeated the movement, this time retrieving the object from the target location and returning it to L_0_ (“retrieve” task: T2). Subjects were instructed not to use compensatory strategies such as torso movements during reaching movements.

Three trials were recorded for each of the five target locations. Target order was randomized for each subject.

### Data processing

#### Joint angle

From the motion capture data, joint angles for 11 DOFs were computed. For the thumb, flexion angle at the IP, MCP, and CMC joints, denoted by IP_1F_, MCP_1F_, and CMC_1F_, respectively, and the abduction angle at the CMC joint (CMC_1A_) were computed. For the fingers, flexion angles at the MCP and PIP joints of the index and middle fingers were computed and averaged across the two fingers to yield MCP_23F_ and PIP_23F_. For the arm, elbow flexion (EL_*F*_), forearm supination (FA_*S*_), shoulder flexion (SH_*F*_), and shoulder abduction (SH_*A*_) were estimated from marker data.

#### Muscle activation

Electromyography signals recorded from the seven target muscles were rectified and low-pass filtered at 5 Hz to create activation profiles. These activation profiles were then normalized by the EMG at maximum contraction to compute normalized activation level of these muscles.

#### Intermuscular coherence

The intermuscular coherence was computed between the following distal and proximal muscles: distal muscles: EDC, FDS, and FDI; proximal muscles: BB, TB, AD, and LD, resulting in 12 pairs. Time-varying coherence values were computed between these muscle pairs to examine the degree of distal-proximal neural coupling throughout the entire movements. For each distal-proximal muscle pair, the time-varying EMG-EMG coherence values were estimated from the raw EMG values using a sliding window of duration 500 ms with an increment of 100 ms, within the MATLAB environment (MathWorks, Inc., Natick, MA, US) employing a script by Neurospec (Halliday et al., 1995).^1^

The coherence values above the 95% confidence level were then *z*-transformed to yield values normally distributed with a standard deviation of approximately 1 (Rosenberg et al., 1989). The *z*-transformed coherence was estimated for the frequency bands between 8 and 55 Hz, which include α-, β-, and γ-bands (α-band: 8–12 Hz, β-band: 13–35 Hz, low γ-band, and γ: 36–55 Hz), commonly used to represent common neural inputs associated with corticospinal pathways (Farmer et al., 1993).

#### Muscle activation

Electromyography signals recorded from the seven target muscles were rectified and low-pass filtered at 5 Hz to create activation profiles. These activation profiles were then normalized by the EMG at maximum contraction to compute normalized activation level of these muscles.

#### Time points and periods

**FIGURE 2:**
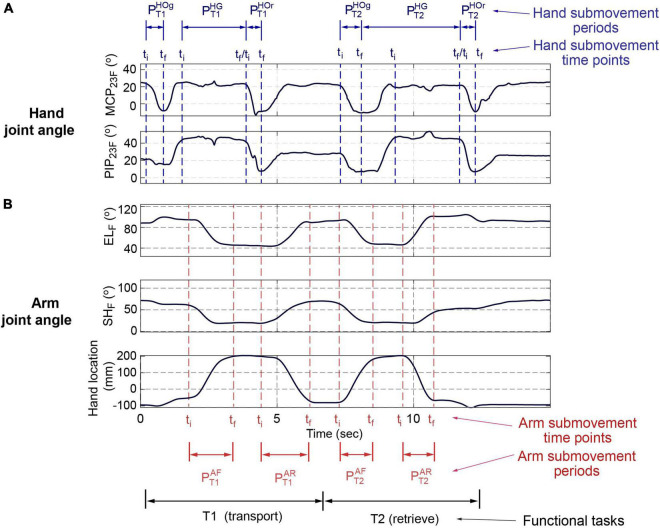
Time points of the UE functional movements (object transport/retrieve; control subject 2; L_3_; trial 2). Here, finger MCP and PIP flexion (MCP_23F_, PIP_23F_) **(A)**, shoulder flexion (SH_F_), elbow flexion (EL_F_), and hand location (in movement direction) **(B)** are shown (MCP, metacarpophalangeal; PIP, proximal interphalangeal). The movement periods for hand movement and arm movement periods are listed in Table 1.

From the angular trajectories of the hand and arm joints, initial and final time points (*t*_*i*_,*t*_*f*_) of the six hand submovement periods, $P_{T\,1}^{H\,O\,g},P_{T\,1}^{H\,G},P_{T\,1}^{H\,O\,r}$ from transport (T1) and $P_{T\,2}^{H\,O\,g},P_{T\,2}^{H\,G},P_{T\,2}^{H\,O\,r}$ from retrieval (T2) (Figure 2A and Table 2A), and the four arm submovement periods, $P_{T\,1}^{A\,F},P_{T\,1}^{A\,R}$ from transport (T1) and $P_{T\,2}^{A\,F},P_{T\,2}^{A\,R}$ from retrieval (T2) (Figure 2B and Table 2B), were identified.

**TABLE 2:** Definition of time points and periods: **(A)** Hand movement periods and time points. **(B)** Arm movement periods and time points.

A						
**Task**	**Period**	**Time point**		**Hand location**	**Submovement**	**Purpose**

Transport (*T_1_*)	$P_{T\,1}^{H\,O\,g}$	*t_i_*	Start of hand open	*L*_0_	Hand: Open (HOg)	To grasp object
		*t_f_*	End of hand open			
	$P_{T\,1}^{H\,G}$	*t_i_*	Start of grip	*L*_0_	Hand: Grasp (HG)	To hold object
		*t_f_*	End of grip	*L_n_*		
	$P_{T\,1}^{H\,O\,r}$	*t_i_*	Start of hand open	*L_n_*	Hand: Open (HOr)	To release object
		*t_f_*	End of hand open			
Retrieve (*T*_2_)	$P_{T\,2}^{H\,O\,g}$	*t_i_*	Start of hand open	near *L_n_*	Hand: Open (HOg)	To grasp object
		*t_f_*	End of hand open			
	$P_{T\,2}^{H\,G}$	*t_i_*	Start of grip	*L_n_*	Hand: Grasp (HG)	To hold object
		*t_f_*	End of grip	*L*_0_		
	$P_{T\,2}^{H\,O\,r}$	*t_i_*	Start of hand open	*L*_0_	Hand: Open (HOr)	To release object
		*t_f_*	End of hand open			

**B**						

**Task**	**Period**	**Time point**		**Hand location**	**Submovement**	**Purpose**

Transport (*T*_1_)	$P_{T\,1}^{A\,F}$	*t_i_*	Start of forward reach	*L*_0_	Arm: Forward reach (AF)	To transport object to target
		*t_f_*	End of forward reach	*L_n_*		
	$P_{T\,1}^{A\,R}$	*t_i_*	Start of retrieval	*L_n_*	Arm: Retrieve (AR)	To return hand to initial position
		*t_f_*	End of retrieval	*L*_0_		
Retrieve (*T*_2_)	$P_{T\,2}^{A\,F}$	*t_i_*	Start of forward reach	*L*_0_	Arm: Forward reach (AF)	To reach for object at target
		*t_f_*	End of forward reach	*L_n_*		
	$P_{T\,2}^{A\,R}$	*t_i_*	Start of retrieval	*L_n_*	Arm: Retrieve (AR)	To return object to initial position
		*t_f_*	End of retrieval	*L*_0_		

### Statistical analysis

#### Proximal-to-distal impact

The hand task kinematics (joint angles), hand muscle activation levels, and the peak intermuscular coherence values were compared between the following hand submovement periods that involved different arm mechanics, using repeated measures analyses of variance (IBM SPSS Statistics; IBM Corp., Armonk, NY, USA). The arm posture (at different time periods) was set as the within-subject variable, and the subject group (controls vs. stroke) as the between-subject variable. For the *post-hoc* pairwise comparisons, the *p*-values were corrected for multiple comparisons with a Bonferroni adjustment.

(1) Distal kinematics: hand aperture (finger extension) and grip posture were compared between the time points that involve different proximal kinematics.
  • Hand aperture for object grip: at near-body (*t*_*f*_ of $P_{T\,1}^{H\,O\,g}$) vs. at target (*t*_*f*_ of $P_{T\,2}^{H\,O\,g}$).
  • Hand aperture for object release: at near-body (*t*_*f*_ of $P_{T\,2}^{H\,O\,r}$) vs. at target (*t*_*f*_ of $P_{T\,1}^{H\,O\,r}$).
  • Grip posture: at near-body (*t*_*i*_of $P_{T\,1}^{H\,G}$) vs. at target (*t*_*i*_of $P_{T\,2}^{H\,G}$).
(2) Distal muscle activation: mean activation level of three hand muscles (EDC, FDS, and FDI) were compared between the following periods with different proximal kinematics.
  • Activation during hand open for object grip: at near-body ($P_{T\,1}^{H\,O\,g}$) vs. at target ($P_{T\,2}^{H\,O\,g}$).
  • Activation during hand open for object release: at near-body ($P_{T\,2}^{H\,O\,r}$) vs. at target ($P_{T\,1}^{H\,O\,r}$).
(3) Intermuscular coherence: peak intermuscular coherence values of the 12 distal-proximal muscle pairs were compared between the following periods.
  • Coherence during hand open for object grip: at near-body ($P_{T\,1}^{H\,O\,g}$) vs. at target ($P_{T\,2}^{H\,O\,g}$).
  • Coherence during hand open for object release: at near-body ($P_{T\,2}^{H\,O\,r}$) vs. at target ($P_{T\,1}^{H\,O\,r}$).
(4) Movement duration: Durations of the following periods (*t_f_-t_i_*) were compared.
  • Duration of hand open for object grip: at near-body ($P_{T\,1}^{H\,O\,g}$) vs. at target ($P_{T\,2}^{H\,O\,g}$).
  • Duration of hand open for object release at near-body ($P_{T\,2}^{H\,O\,r}$) vs. at target ($P_{T\,1}^{H\,O\,r}$).

#### Distal-to-proximal impact

The arm kinematics (joint angles) and arm muscle activation levels were compared between the following arm submovement periods that involved different hand mechanics, using repeated measures analyses of variance. The distal task (hand open vs. grip) at different periods was set as the within-subject variable, and the subject group (controls vs. stroke) as the between-subject variable. Again, for the *post-hoc* analysis, the *p*-values were corrected with a Bonferroni adjustment.

(1) Proximal kinematics: arm joint angles were compared between the following conditions (two distal tasks).
  • Arm posture at target (L_*n*_): with grip (*t*_*f*_ of $P_{T\,1}^{A\,F}$) vs. with hand open (*t*_*f*_ of $P_{T\,2}^{A\,F}$).
(2) Proximal muscle activation: mean activation level of four arm muscles (BB, TB, AD, and LD) were compared between the following conditions.
  • Mean activation during reaching: with grip ($P_{T\,1}^{A\,F}$) vs. with hand open ($P_{T\,2}^{A\,F}$).
(3) Movement duration: Durations of the following periods (*t_f_-t_i_*) were compared.
  • Duration of reaching: with grip ($P_{T\,1}^{A\,F}$) vs. with hand open ($P_{T\,2}^{A\,F}$).

## Results

Inability to open their hands prevented a subset of stroke survivors (S1–S3) from voluntarily completing the task; three subjects with severe UE impairments could not voluntarily perform the task, as they failed to extend the thumb/fingers enough to grip the object at the near-body location (L_0_). Thus, only their proximal kinematics and EMG data were included in the analysis. Six stroke survivors with moderate impairment and five neurologically intact control subjects were able to complete the tasks, and all of their data were used for consequent analyses.

### Impact of proximal mechanics on distal task mechanics

**FIGURE 3:**
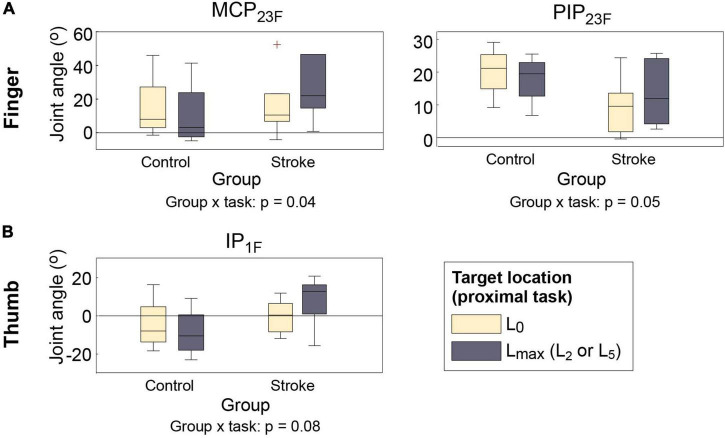
Distal kinematics of two subject groups under two proximal task conditions ($P_{T\,1}^{H\,O\,r}$ vs. $P_{T\,2}^{H\,O\,r}$). **(A)** Finger MCP (MCP_23F_); **(B)** finger PIP (PIP_23F_); **(C)** thumb IP (IP_1F_) joint flexion angles. For each box, the central mark indicates median, and the bottom and top edges of the box indicates 25th and 75th percentiles, respectively (MCP, metacarpophalangeal; PIP, proximal interphalangeal). The whiskers extend to the most extreme data points excluding outliers (which were indicated by “+” symbols).

Across participants, movements of two proximal DOFs (elbow extension, shoulder abduction) significantly affected distal movements, particularly the maximum hand aperture for object grip (i.e., with no object in hand: $P_{T\,1}^{H\,O\,g}$ vs. $P_{T\,2}^{H\,O\,g}$). The responses of the two groups, however, were very different. When opening the hand to grasp the object at the target locations (during $P_{T\,2}^{H\,O\,g}$), the hand aperture of stroke survivors became significantly smaller than their aperture at near-body location (L_0_) (during $P_{T\,1}^{H\,O\,g}$), as their finger and the thumb joint flexion angles increased, and the difference was the largest at the target locations that required shoulder abduction (L_5_) or elbow extension (L_2_). Control subjects, in contrast, increased their hand aperture at these target locations with respect to the near-body location L_0_ (group × proximal task interaction; *p* = 0.04 for MCP_23_; *p* = 0.05 for PIP_23_, *p* = 0.08 for IP_1_; Figure 3). A similar group × proximal task interaction was observed during hand opening periods for object release (during $P_{T\,1}^{H\,O\,r}$ vs. during $P_{T\,2}^{H\,O\,r}$) but the difference was smaller, and reached statistical significance only for one thumb joint (IP_1_; *p* = 0.02).

In contrast, no significant differences in grip posture were found across locations.

**FIGURE 4:**
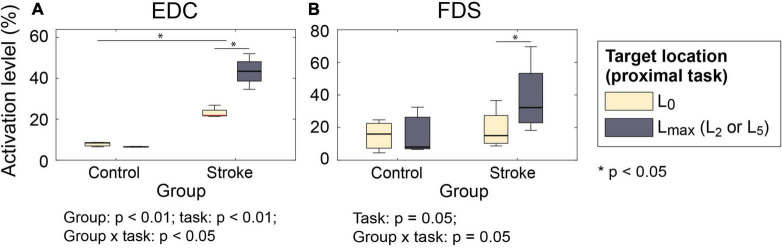
Muscle activation level during hand open phase under two proximal task conditions ($P_{T\,1}^{H\,O\,r}$ vs. $P_{T\,2}^{H\,O\,r}$). **(A)** EDC; **(B)** FDS. For both muscles, the increase in the activation level was significantly greater for stroke survivors (group × proximal task: *p* < 0.01 for EDC and *p* = 0.05 for FDS) (EDC, extensor digitorum communis; FDS, flexor digitorum superficialis).

Similar group × proximal task interactions were observed in the muscle activations. When two hand open movements were compared (near-body vs. at target locations L_2_/L_5_; $P_{T\,1}^{H\,O\,g}$ vs. $P_{T\,2}^{H\,O\,g}$), activation level of both extrinsic hand muscles (EDC and FDS) during hand opening prior to grip significantly increased in stroke survivors at the target locations requiring shoulder abduction/elbow extension (L_2_/L_5_) (EDC: 21.4–26.8% at L_0_ vs. 34.6–52.0% at L_2_/L_5_; FDS: 7.9–17.3% at L_0_ vs. 10.6–33.0% at L_2_/ L_5_), but not in control subjects (group × proximal task: *p* < 0.01 for EDC; *p* = 0.05 for FDS; Figure 4). Similar trends were found in the activation of the FDI and thenar muscles, but to a lesser degree (*p* > 0.07).

**FIGURE 5:**
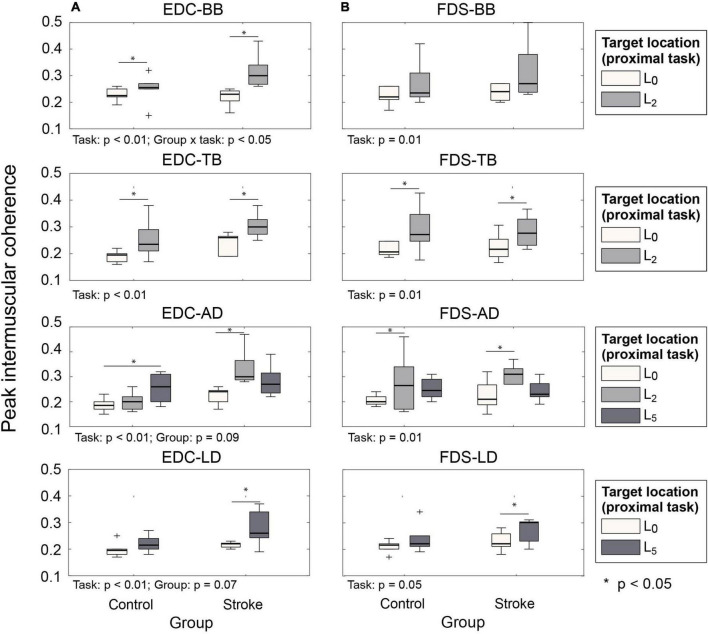
Peak intermuscular coherence value during hand open phase under two proximal task conditions ($P_{T\,1}^{H\,O\,r}$ vs. $P_{T\,2}^{H\,O\,r}$). **(A)** EDC and proximal muscle pairs. **(B)** FDS and proximal muscle pairs (EDC, extensor digitorum communis; FDS, flexor digitorum superficialis).

**FIGURE 6:**
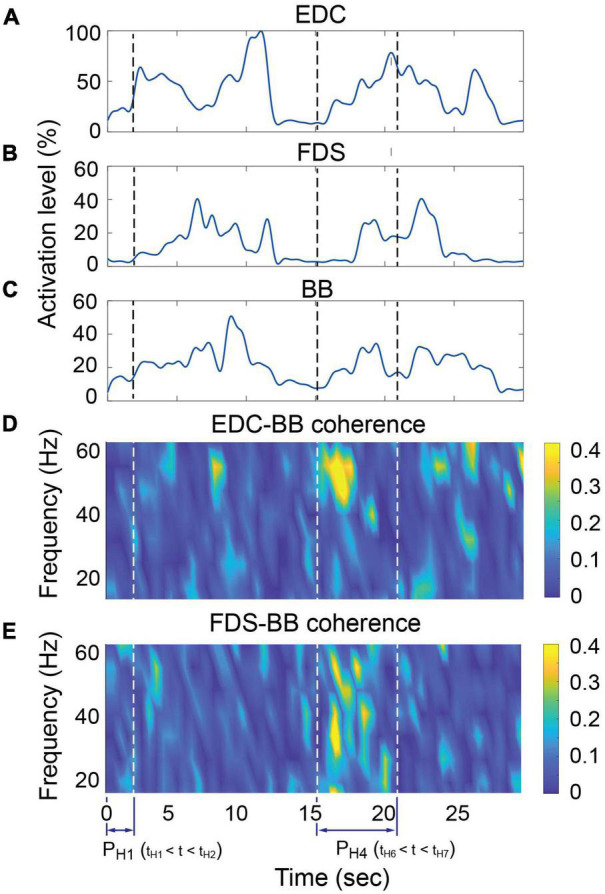
Representative case of muscle activation and intermuscular coherence during task (Stroke subject 4, location 2, trial 1). **(A–C)** Activation profile of EDC, FDS, and BB. **(D–E)** Time-frequency plot of intermuscular coherence of EDC-BB and FDS-BB pairs. Increase in the intermuscular coherence in the β- (13–35 Hz) and low γ- (36–55 Hz) bands was mainly observed during early phase of the reaching movements toward the target (i.e., the first half of P_H3_), which was followed by an increase in the activation of the distal muscles (EDC and FDS). Such patterns were not observed at near-body location (i.e., during P_H1_) (EDC, extensor digitorum communis; FDS, flexor digitorum superficialis; BB, Biceps brachi).

Intermuscular coherence between the hand muscles (EDC, FDS, and FDI) and the agonist/antagonist muscles of each proximal movement (i.e., BB/TB/AD for L_2_; LD for L_5_) was significantly higher at the target locations away from the body than the near body location (L_0_) for both subject groups (Figure 5). During the hand open periods at target locations (L_2_ or L_5_, i.e., during approach to retrieve), coherence between distal and proximal muscles increased at the early phase of the reaching movements, which was followed by the increase in the activation of the distal muscles (EDC and FDS; during $P_{T\,2}^{H\,O\,g}$, Figure 6). Such an increase in cocontraction during the hand open phase was not observed when the hand was near the body (during $P_{T\,1}^{H\,O\,g}$, Figure 6).

### Impact of distal mechanics on proximal task mechanics

The type of distal movements (hand open vs. grip) also significantly affected coordination of proximal DOFs (shoulder abduction and elbow extension) during outward movements (i.e., reaching–$P_{T\,1}^{A\,F}$: transport/reaching with grip vs. $P_{T\,2}^{A\,F}$: approach/reaching with hand open). The largest difference in proximal kinematics was observed in the target locations that required shoulder abduction (L_3_ and L_5_).

**FIGURE 7:**
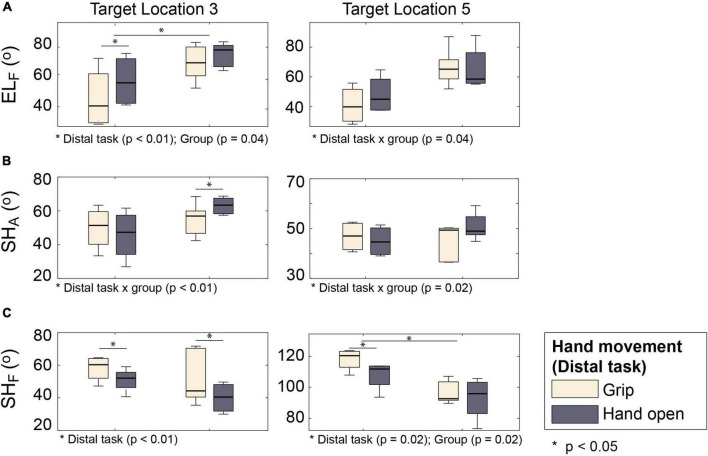
Proximal kinematics of two subject groups under two distal task conditions ($P_{T\,1}^{A\,F}$ vs. $P_{T\,2}^{A\,F}$). **(A)** Elbow flexion (EL_F_). **(B)** Shoulder abduction (SH_A_). **(C)** Shoulder flexion (SH_F_).

For some proximal DOFs, similar patterns of distal-to-proximal impact were observed in both subject groups: subjects adopted postures with greater elbow flexion (*p* < 0.01) (Figure 7A) and smaller shoulder flexion (*p* < 0.01) during approach ($P_{T\,2}^{A\,F}$: distal task–hand open) than transport ($P_{T\,1}^{A\,F}$: distal task: grip) (Figure 7C).

The distal-to-proximal impact on other proximal DOFs, however, differed between the groups (i.e., significant group × distal task interaction). Shoulder abduction of stroke survivors was greater during approach ($P_{T\,2}^{A\,F}$: hand open) than during transport ($P_{T\,1}^{A\,F}$: grip), while that of control subjects was similar during these two tasks (Figure 7B; group × distal task *p* < 0.01). Conversely, the difference in elbow flexion between approach ($P_{T\,2}^{A\,F}$: with hand open) and transport ($P_{T\,1}^{A\,F}$: with grip) was greater for control subjects than for stroke survivors (Figure 7A; group × distal task *p* = 0.06).

When the arm muscle activation patterns were compared between the two hand movements (hand open vs. grip), however, there was no significant between-task differences in the activation level of most arm muscles, except for the elbow extensor muscle (TB), which exhibited a higher activation level when reaching with hand open (*p* = 0.01). No significant group differences or group × distal task interactions were found.

### Impact of distal-proximal coupling on temporal aspect of task performance

**FIGURE 8:**
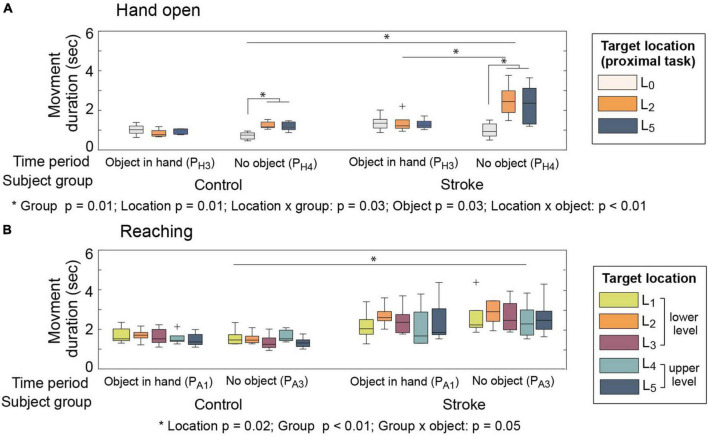
Movement duration. **(A)** Hand movement (hand open). **(B)** Arm movement (reaching).

Movement time of stroke survivors was significantly longer than that of control subjects for both distal (hand open) and proximal (reaching) movements (both *p*-values ≤0.01) (Figures 8A,B).

A similar impact of proximal movements on the movement duration of distal segments was observed in both subject groups. Hand open duration was longer when subjects approach the object ($P_{T\,2}^{H\,O\,g}$) than when they released it ($P_{T\,1}^{H\,O\,r}$) (object: *p* = 0.03), and the amount of increase in movement duration was greater at certain target locations (L_2_ and L_5_) when compared to the near-body location (L_0_) (proximal task × object: *p* = 0.01). Similar trends (i.e., proximal task—object interaction) were observed in both groups, although the amount of increase in movement duration due to proximal task was greater for stroke subjects (Figure 8A) (group × proximal task × object: *p* = 0.08).

The impact of distal movements on the proximal movement duration, however, was different between the subject groups. Reaching movements of stroke survivors lasted longer when they approached the object ($P_{T\,2}^{A\,F}$; distal task—hand open) than when they transported the object ($P_{T\,1}^{A\,F}$; distal task—grip), while the movement duration of healthy subjects was similar between these two phases ($P_{T\,1}^{A\,F}$ vs. $P_{T\,2}^{A\,F}$) (group × object: *p* = 0.05; Figure 8B).

## Discussion

### Deficits in finger/thumb extension movements of stroke survivors

Deficits in the ability to extend the fingers and thumb are commonly observed after stroke (Kamper and Rymer, 2001; Kamper et al., 2003, 2006; Lang et al., 2009). This reduced digit extension is thought to significantly contribute to overall functional impairment of stroke survivors. Active finger extension of stroke survivors in the early phase of recovery is predictive of eventual recovery of overall upper extremity function (Smania et al., 2007; Stinear et al., 2012) or recovery after movement therapy (Fritz et al., 2005).

Our results highlighted the importance of finger/thumb extension in functional task performance of stroke survivors. In accordance with our hypothesis, we found a significant proximal-to-distal impact (which was stronger than the distal-to-proximal impact), mainly observed during the tasks that involved hand opening. For the stroke survivors with severe impairments (Fugl-Meyer UE score <25), an inability to open their hands prevented them from performing the reach-transport-release task, as they were unable to grip the object. This is consistent with previous studies that showed successful grip of objects by stroke survivors can only be achieved when they restore their ability to extend the fingers and thumb (Lang et al., 2009).

Even for the participants with moderate impairment, although they were able to complete the task, significant changes in the task mechanics were observed, especially during movements requiring finger/thumb extension. First, the duration of the hand opening phase was found to be significantly longer than that for neurologically-intact subjects. In contrast, the difference between the two groups in movement duration while gripping (transporting) the object was much smaller. Unlike neurologically-intact subjects, the hand aperture of stroke survivors during retrieval was significantly smaller for distant targets than for the near-body locations, likely contributing to the observed delays in hand openings. Finger/thumb extension of stroke survivors also affected their proximal mechanics; the shoulder abduction of stroke survivors during object approach (with hand open) was significantly greater than that during object transport (with grip).

### Functional impact of proximal mechanics on distal task performance and its underlying mechanism

Proper adjustment of hand aperture during object retrieval is an important motor skill developed over time (Kuhtz-Buschbeck et al., 1998). In neurologically intact individuals, hand aperture is adjusted by visual feedback; when visual information of the object (size/shape) is unavailable, hand aperture is increased (exaggerated hand opening) to deal with the uncertainty (Jakobson and Goodale, 1991). Hand aperture during retrieval also needs to be increased to compensate for potential directional errors in reaching movements, which affect hand placement with respect to the object (Bootsma et al., 1994).

In accordance with the previous studies, in our experiments, control subjects increased their hand aperture during retrieval of the object at the target locations (farther from the body), possibly to compensate for potential directional errors in reaching movements or due to reduced visual information of the hand aperture due to the distance. Stroke survivors, however, had decreased hand aperture when retrieving objects from target locations that require shoulder abduction or elbow extension. This decrease in aperture could have significantly hampered their ability to place/orient the hand to be able to grip the object, as well as increasing the task duration.

The observed reduction in the hand aperture of stroke survivors at these target locations resulted from global increases in the activation levels of the hand muscles, particularly those of the extrinsic muscles. As the cross-sectional areas of the extrinsic flexor muscles (e.g., flexor digitorum profundus and FDS) are much greater than those of extrinsic extensor muscles (EDC or extensor indicis proprius) (Lieber et al., 1992; Valero-Cuevas et al., 1998), equal coactivation of extrinsic finger muscles would produce a net flexion. Substantial activation of extensor muscles is needed to counteract even a moderate increase in activation of flexor muscles; this increase may be challenging for stroke survivors to achieve as they often have limited capacity to activate their extrinsic finger extensor muscles (Hoffmann et al., 2016). Indeed, activation of both extrinsic hand muscles, EDC (extensor) and FDS (flexor), increased to a similar degree as stroke survivors opened their hands at these target locations (mean ± SD change in activation from L_0_ to L_2_/L_5_: EDC = 20.4 ± 7.8%, FDS = 19.2 ± 22.8%), thereby resulting in the fingers and thumb being more flexed when reaching the target, in a manner similar to the typical flexion synergy patterns observed in stroke survivors (Brunnstrom, 1970). In contrast, no significant changes were observed in the muscle activation pattern of control subjects (mean ± SD change in activation from L_0_ to L_2_/L_5_: EDC = 1.3 ± 1.2%, FDS = 0.6 ± 9.0%).

The increase in the activation levels of the hand muscles appears to be related to the increase in the intermuscular coherence at these target locations. Previous studies observed that significant increase in the intermuscular coherence of the hand muscles was accompanied by a concurrent increase in all muscle activation levels (i.e., higher cocontraction) (Danna-Dos Santos et al., 2010; Vermillion et al., 2015). Abnormal antagonist muscle cocontraction in other neurological conditions (i.e., dystonia) was also linked to the increased intermuscular coherence (Farmer et al., 1998). Thus, our results suggest that the increase in the hand muscle activation level at the target locations that require shoulder abduction/elbow extension could be explained by the increased neural coupling between the hand (EDC and FDS) and the arm (BB, TB, AD, and LD) muscles.

### Impact of distal movements on proximal task performance

A significant distal-to-proximal impact on task mechanics was also observed across groups, as subjects extended their elbow further during object release ($P_{T\,1}^{A\,R}$). Stroke survivors also abducted their shoulder as they approached the object ($P_{T\,2}^{A\,F}$). However, unlike the proximal-to-distal impact, no significant group differences or group × task interactions were found in the activation level of the arm muscles.

Therefore, the observed group × distal task interactions in the proximal kinematics (elbow flexion and shoulder abduction) appear to be different from the proximal-to-distal effects, which resulted from a significant increase in distal muscle cocontraction. The observed changes in the proximal movement pattern of stroke survivors (e.g., increased shoulder abduction during the hand open phase) is likely due to their compensatory strategy. For instance, during approach (distal task: hand open), it was observed that some stroke survivors had to move their hands laterally to grip the object from the side due to the decreased hand aperture. This required greater shoulder abduction, which could lead to further hand closing in accordance with abnormal flexion synergy patterns (i.e., elbow flexion-shoulder abduction; Brunnstrom, 1970).

### Implications

Our study emphasizes the interactions between proximal and distal limb movements in UE functional activities performed by stroke survivors. The ability to extend the fingers and thumb is a prerequisite to object transport, as stroke survivors who could not open their hands were not able to initiate the transport. Even for those participants who were able to perform the task, proximal arm postures and motions significantly affected finger/thumb extension (reduced hand aperture), while deficits in hand opening required compensatory changes in proximal kinematics. Therefore, restoration of the ability to extend the impaired fingers and thumb should be a primary focus for stroke rehabilitation, even for those who retain the ability to open the hand. More importantly, hand opening should be practiced in conjunction with proximal (reaching) movements so that stroke survivors can learn to overcome the abnormal distal-proximal coupling that affects their task performance.

### Limitations of the study

The number of subjects in this pilot study was small as we aimed to test feasibility of the detailed analysis of kinematic/physiological data throughout the complex upper extremity functional task. Therefore, the results from this study need to be replicated in a future study with a larger number of subjects in order for its findings to be generalized. The small subject number could also have led to type-II errors in the changes in the kinematic (EMG) and physiological (muscle activation) measures, whose *p*-values were greater than 0.05. Observational studies are required to examine the impact of abnormal proximal-distal interactions in daily activities of stroke survivors.

Between-subject variability in the impairment of stroke survivors could have contributed to the variability in the data. The level of spasticity of stroke subjects, as well as the degree of their sensory deficits, was neither measured nor controlled, which could have contributed to the between-subject variability observed in the data.

The control subjects were much younger than the stroke survivors tested in this study, which could have contributed to the observed between-group differences, in particular the movement time. However, previous studies showed that, while older subjects make slower movements during reaching, their movement coordination patterns remain similar to that of younger subjects (Bennett and Castiello, 1994).

Some stroke subjects adopted compensatory strategies, involving torso movements (mainly tilting and twisting, since the torso was strapped to the chair), during reaching movements. Experimenters instructed subjects not to use such strategy, whenever detected, but it is possible that stroke survivors used torso movements to a certain degree during reaching; this could have reduced the requirements in their arm extension, or could have affected the hand orientation.

## Conclusion

In this pilot study, we delineated how abnormal coupling between the movements of distal and proximal UE segments of chronic stroke survivors affect their functional movements. In particular, hand open (distal) and elbow extension and shoulder abduction (proximal) movements influence each other, resulting in a significant reduction in the distal range of motion (finger extension) and an abnormal proximal synergy pattern (increased elbow flexion/shoulder abduction) that can degrade task performance. Increased cocontraction of the hand muscles due to higher neural couplings between the distal and proximal muscles, indicated by the significant increase in the intermuscular coherence, appears to be the underlying mechanism of the observed functional deficits. The outcome of this study suggests that the finger/thumb extension of chronic stroke survivors should be practiced in conjunction with specific proximal movements (elbow extension/shoulder abduction) to help them overcome the abnormal distal-proximal coupling affecting task performance.

## Data availability statement

The raw data supporting the conclusions of this article will be made available by the authors, without undue reservation.

## Ethics statement

The studies involving human participants were reviewed and approved by the Institutional Review Boards of MedStar Health Research Institute. The patients/participants provided their written informed consent to participate in this study.

## Author contributions

SL and BV designed the study. TP, HN, and BV collected the data. TP, HN, and SL analyzed the data. All authors interpreted the data, wrote the manuscript, and read and approved the final manuscript.

## Footnotes

1
www.neurospec.org
